# Assessing Preferences of Facial Appearance in Transgender and Gender Nonbinary Patients

**DOI:** 10.1007/s00266-023-03715-2

**Published:** 2023-11-07

**Authors:** Brendan J. Cronin, Sarah Fadich, Justine C. Lee

**Affiliations:** Division of Plastic and Reconstructive Surgery, Department of Surgery, UCLA David Geffen School of Medicine, University of California, Los Angeles, 200 UCLA Medical Plaza, Suite 460, Los Angeles, CA 90095 USA

**Keywords:** Facial feminization surgery, Facial masculinization surgery, Aesthetic preferences, Nonbinary, Transgender, Transgender female, Transgender male, Gender affirming facial surgery

## Abstract

**Background:**

We designed a survey to evaluate preferences of facial appearance in transgender male (TM), transgender female (TF) and gender nonbinary patients to better inform goals of facial gender affirming surgery (FGAS) in gender nonbinary patients.

**Methods:**

TM/TF and nonbinary patients > 18 years old were identified via retrospective chart review and distributed an anonymized survey via email from October 3 to December 31, 2022. To assess facial preferences, AI-generated and open-source portraits were edited to create five image sets with a range of features from masculine to feminine for the forehead, mandible/chin and hairline. Data were analyzed using Fisher’s exact tests and ANOVA in R-Studio.

**Results:**

Survey response rate was 32% (180 patients identified via chart review, 58 respondents; TM = 5, TF = 39, nonbinary = 14). TM and TF patients as well as TF and nonbinary patients had significantly different preferences for all regions (*p* < 0.005; all series), while TM and nonbinary patients did not (*p* => 0.05; all series). TF patients consistently selected 4s with neutral or more feminine features. TM and nonbinary patients, however, demonstrated no consistent preference for either male or female features but rather a range of responses spanning extremes of both masculine and feminine options. When stratified by sex assigned at birth, nonbinary patients consistently identified preferences opposite to their assigned gender.

**Conclusion:**

Gender nonbinary and TM patients appear to have uniquely individual preferences regarding facial appearance that do not fit into classically masculine or feminine patterns/phenotypes. As a result, we recommend individualized preoperative planning for FGAS to achieve the optimal result in these patient populations.

**Level of Evidence IV:**

This journal requires that authors assign a level of evidence to each article. For a full description of these Evidence-Based Medicine ratings, please refer to the Table of Contents or the online Instructions to Authors www.springer.com/00266.

## Introduction

Facial gender-affirming surgery (FGAS) entails the crafting of a masculine or feminine appearance from a patients’ underlying craniofacial skeleton and soft tissue structures to alleviate dysphoria secondary to facial characteristics. As the endeavor involves transformation of anatomy of one gender to anatomy of a different gender, average and "ideal" anatomic relationships for feminine and masculine faces—and the key differences between the two—are fundamental to obtaining satisfactory and aesthetic outcomes. Anthropometric studies by Farkas and Ousterhout, among others, have previously described these relationships [1–8]. Secondary to these works, FGAS in binary transgender patients identifying as transgender male/transgender female has specific defined targets for appearance of various facial areas [6, 9–11].

While nonbinary patients may have dysphoria with their physical facial characteristics [12], they may not desire a uniformly masculine or feminine facial appearance. As a result, the preferences of this patient population and therefore goals of FGAS in nonbinary patients are less well defined. In order to assess this, we performed a cross-sectional survey of transgender and nonbinary patients that evaluated preferences of facial appearance on a series of images ranging from "masculine" to "feminine" features of key gender-defining facial regions. We hypothesized transgender male (TM) and transgender female (TF) patients would elect specifically feminine or masculine features, whereas nonbinary patients would elect images spanning the full range of masculine to feminine features.

## Methods

### Patient Identification and Inclusion Criteria

A retrospective chart review was performed from July 2018 to September 2022 to identify patients for survey distribution. Patients were included if they presented for gender-affirming surgical consultation, were greater than 18 years of age, identified as transgender or nonbinary and had email contact information available in the medical record. Prior to conducting this study, approval was obtained from our institution review board (IRB).

### Survey Creation

A 26-question, multiple-choice survey, was designed using the Qualtrics survey platform (Qualtrics, Seattle, WA). The survey collected data on participant demographics, gender transition history, history of prior medical or surgical interventions for gender dysphoria, and preferences of facial features as described below. The survey was distributed via email with an anonymized link such that all responses were collated without identifiable information.

### Image Generation

Survey images were designed to (1) focus on facial features/regions that most significantly contribute to a masculine or feminine appearance [6, 8, 9] and (2) represent a realistic spectrum of these features from "most masculine" to "most feminine" as based on existing anthropometric studies [1–3, 6, 10, 11, 13]. As a result, we chose to focus on the appearance of the forehead (frontal view, lateral view), hairline pattern (3/4 view), and mandible/chin (frontal view, lateral view). Five separate image series were generated to isolate the impact of the change in appearance of a certain facial region without distracting the viewer with changes in other facial features.

**Fig. 1:**
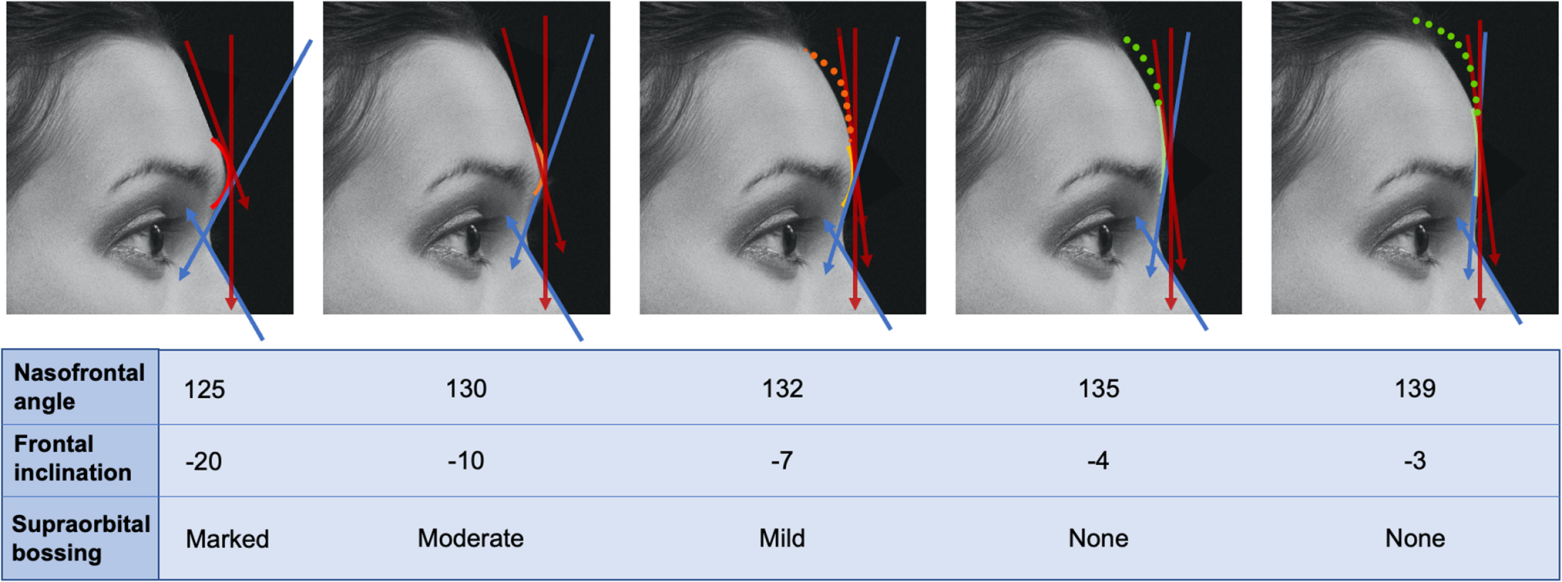
Nasofrontal complex image schematics. An open-source profile-view portrait was edited to change the nasofrontal angle, frontal inclination and degree of supraorbital bossing from ranges derived from anthropometric studies for "masculine" and "feminine" facial ideals/appearances. The image series above demonstrates the original photograph with the planned edits overlayed in red (frontal inclination), blue (nasofrontal angle) and multi-colored arcs (frontal bossing)

A database of artificial-intelligence generated photographs (Generated.photos, Generated Media, Inc) was reviewed to identify gender neutral appearing portraits for manipulation with photoshop (Photoshop, Adobe; San Jose, CA). Open-source images were also reviewed as AI generated images were only available for frontal and 3/4 profile views. Series were designed with a middle "neutral" image flanked by progressively "masculine" or "feminine" images on either side. When applicable, data from anthropometric studies (i.e., forehead inclination [1, 13], nasofrontal angle [14–17], gonial angle [18]) were used to define the extremes and "ideals" of the masculine/feminine images. Similarly, standard deviation of these measures guided the transition of features across each respective image series [14–18]. Details on the design of the various image series are described below (Fig. 1).

### Frontal Forehead and Hairline Oblique

**Fig. 2:**
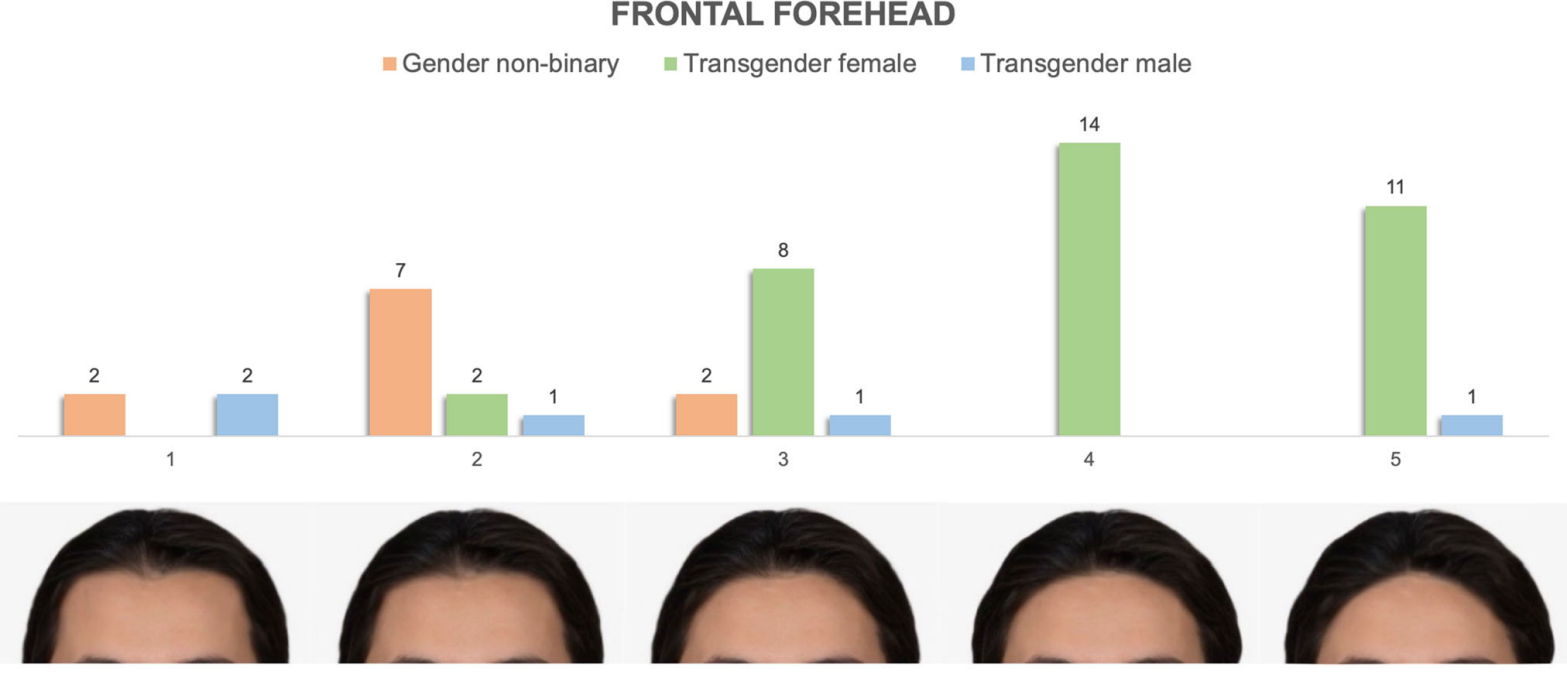
Preferences of frontal forehead appearance stratified by gender identity. It demonstrates tabulated survey responses (stratified and color-coded by gender identity) above their corresponding images, numbered 1–5. The images in the frontal forehead view series range from masculine “M” and “rectangular”-shaped, with long, non-hair bearing forehead height, broader intertemporal distance and frontotemporal recessions to more feminine appearance foreheads typified by shorter forehead height, narrower width, and round, "bell-shaped" or apex/triangular hairline shape

**Fig. 3:**
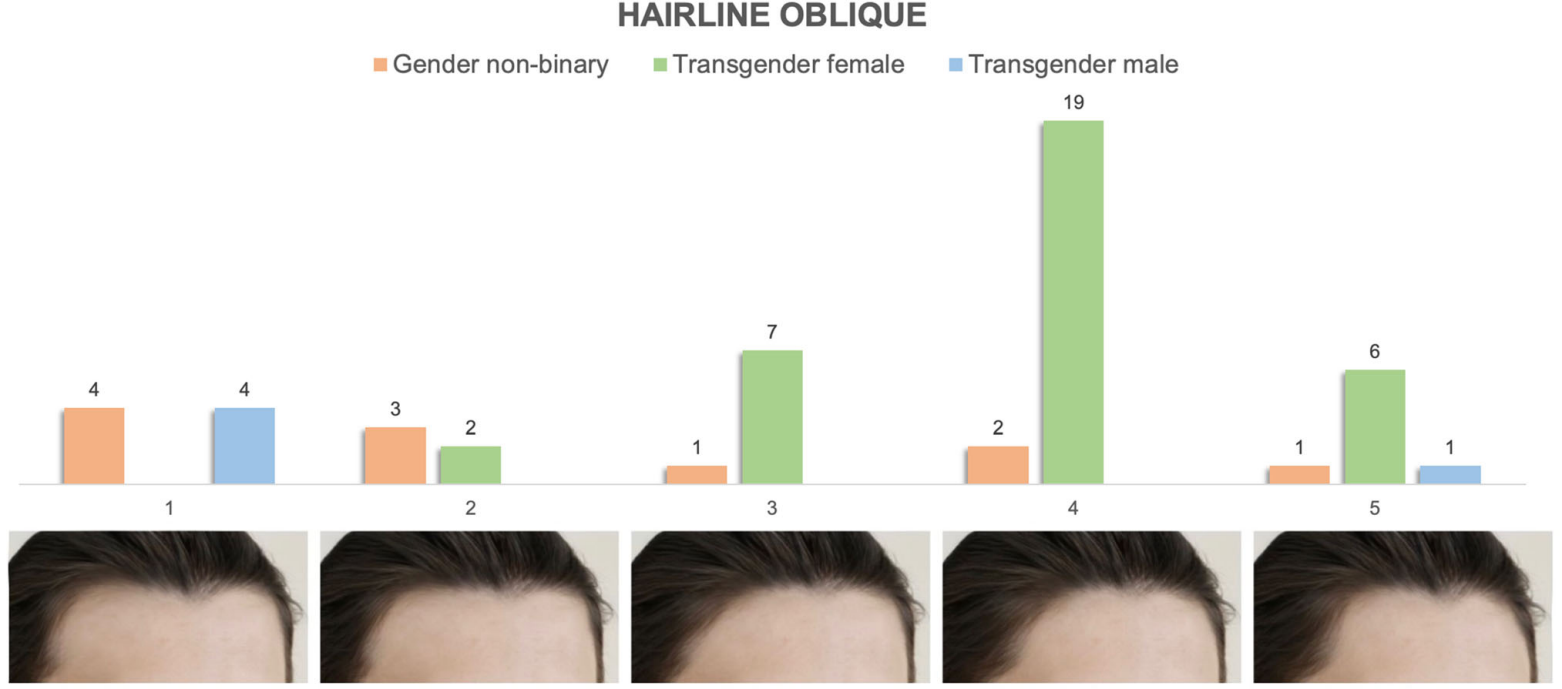
Preferences of hairline (oblique forehead) appearance stratified by gender identity. It demonstrates tabulated survey responses (stratified and color-coded by gender identity) above their corresponding images, numbered 1–5. The images in the oblique forehead view series range from a broad, tall masculine forehead with frontotemporal recession to shorter, narrower feminine foreheads with either no frontotemporal recess or the presence of temporal points

Forehead height, shape and hairline pattern are classically described differences in masculine and feminine facial appearance that can be modified to varying degrees during FGAS [6, 9]. The images in our frontal forehead view series range from masculine “M” and rectangular-shaped, with long, non-hair bearing forehead height, broader intertemporal distance and frontotemporal recessions to more feminine appearing foreheads typified by shorter forehead height, narrower width, and round, “bell-shaped” or apex/triangular hairline shape [6, 9, 19–21] (Fig. 2). On oblique view (Fig. 3), which offers a better view of the frontotemporal area of the hairline, our series ranges from broad, tall masculine foreheads with frontotemporal recession to shorter, narrower feminine foreheads with either no frontotemporal recess or the presence of temporal points [19–21].

### Lateral Forehead

**Fig. 4:**
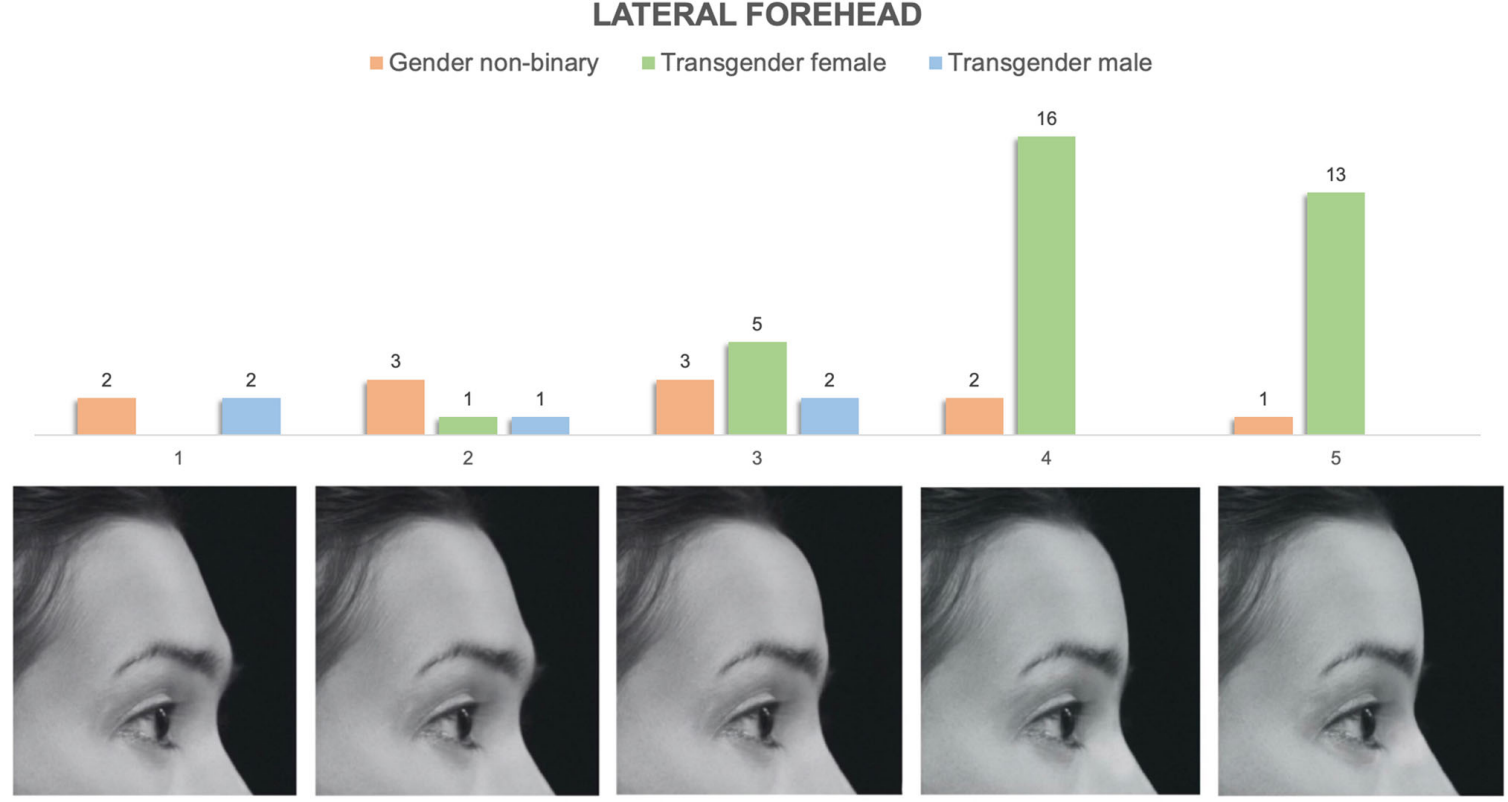
Preferences of lateral forehead (nasofrontal complex) appearance stratified by gender identity. It demonstrates tabulated survey responses (stratified and color-coded by gender identity) above their corresponding images, numbered 1–5. The images in the lateral forehead series span a range of forehead slope, frontal bossing and nasofrontal angle as described in Fig. 1

The nasofrontal complex represents one of the key areas where the underlying craniofacial skeleton most directly contributes to masculine/feminine appearance. Elements include forehead slope, frontal bossing and nasofrontal angle. A combination of surveys, retrospective CT reviews and classic texts have reported the average and “ideal” masculine and feminine nasofrontal angles to be 130 and 133–140 degrees, respectively, with an “acceptable range” of 120–133 degrees for males and 128–140 for females [13, 15]. Accordingly, our five images span a nasofrontal angle range of 125–139 degrees (Figs. 1, 4). Anthropometric and CT-based studies of forehead retroclination vary slightly in their reported ranges but generally describe average male retroclination of − 7 to − 10 degrees (range 2 to − 23) and average female retroclination of − 3.5 to − 5.5 (range + 6 to − 17) [1–3, 13]. Quantification of frontal bossing has predominantly been based on brow protrusion on clinical examination. Ousterhout described this as 10mm for Caucasians and 6-8mm in East Asians [22]. In data from our institution characterizing globe to forehead distance during clinical examination prior to FGAS, we have encountered ranges from 7 to 20 mm. Given absolute distances are challenging to represent on digital photographs, we segregated frontal bossing into none, mild, moderate and severe (Fig. 1).

### Frontal and Lateral Mandible/Chin

**Fig. 5:**
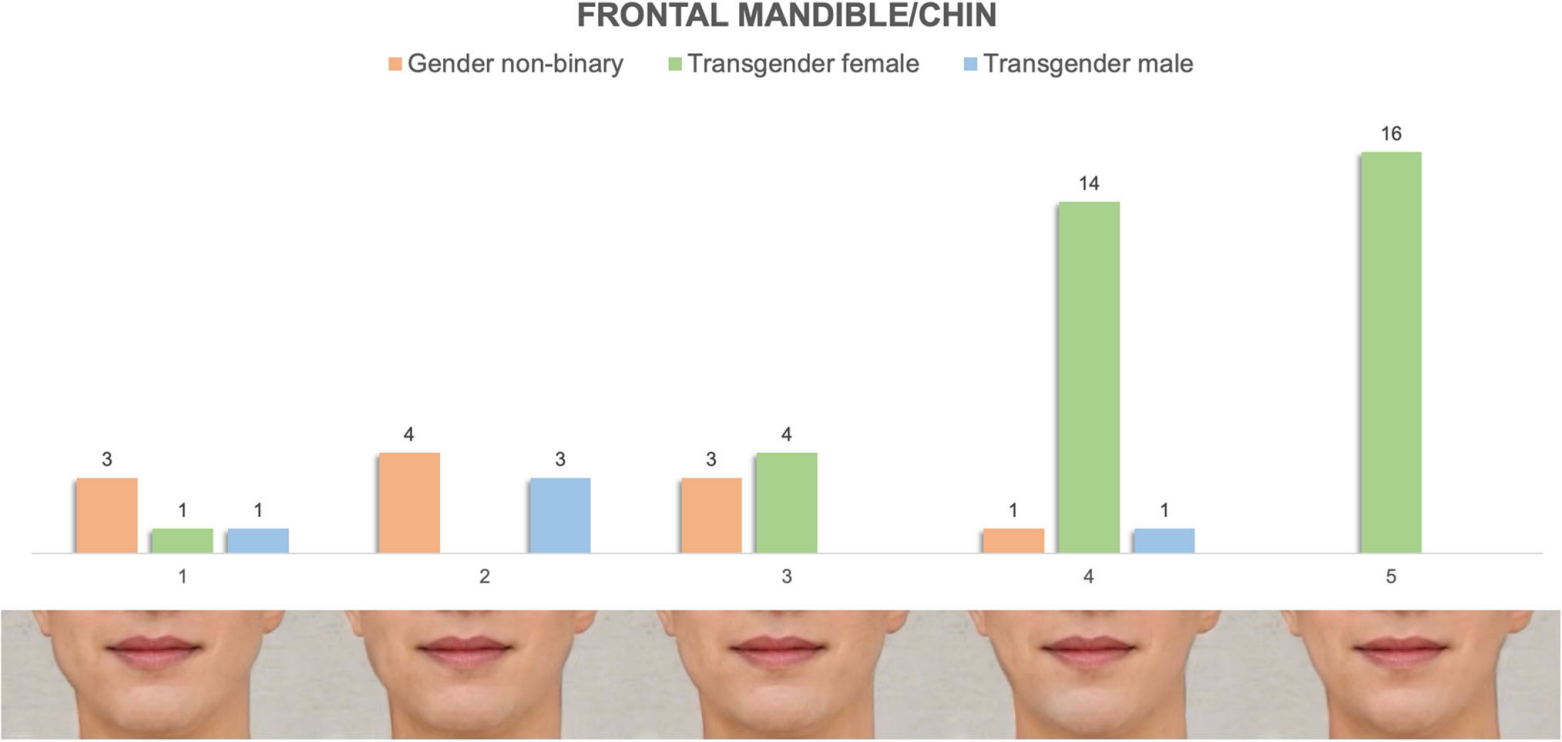
Preferences of frontal mandible/chin appearance stratified by gender identity. It demonstrates tabulated survey responses (stratified and color-coded by gender identity) above their corresponding images, numbered 1–5. On frontal view, feminine images were characterized by narrow intergonial and chin width with a soft, heart-shaped contour of the lower face. Masculine images were characterized by wider and more well-defined gonial angles of 127–130 degrees, taller, wider and more well-defined chins and overall, more angular appearance of the lower face

**Fig. 6:**
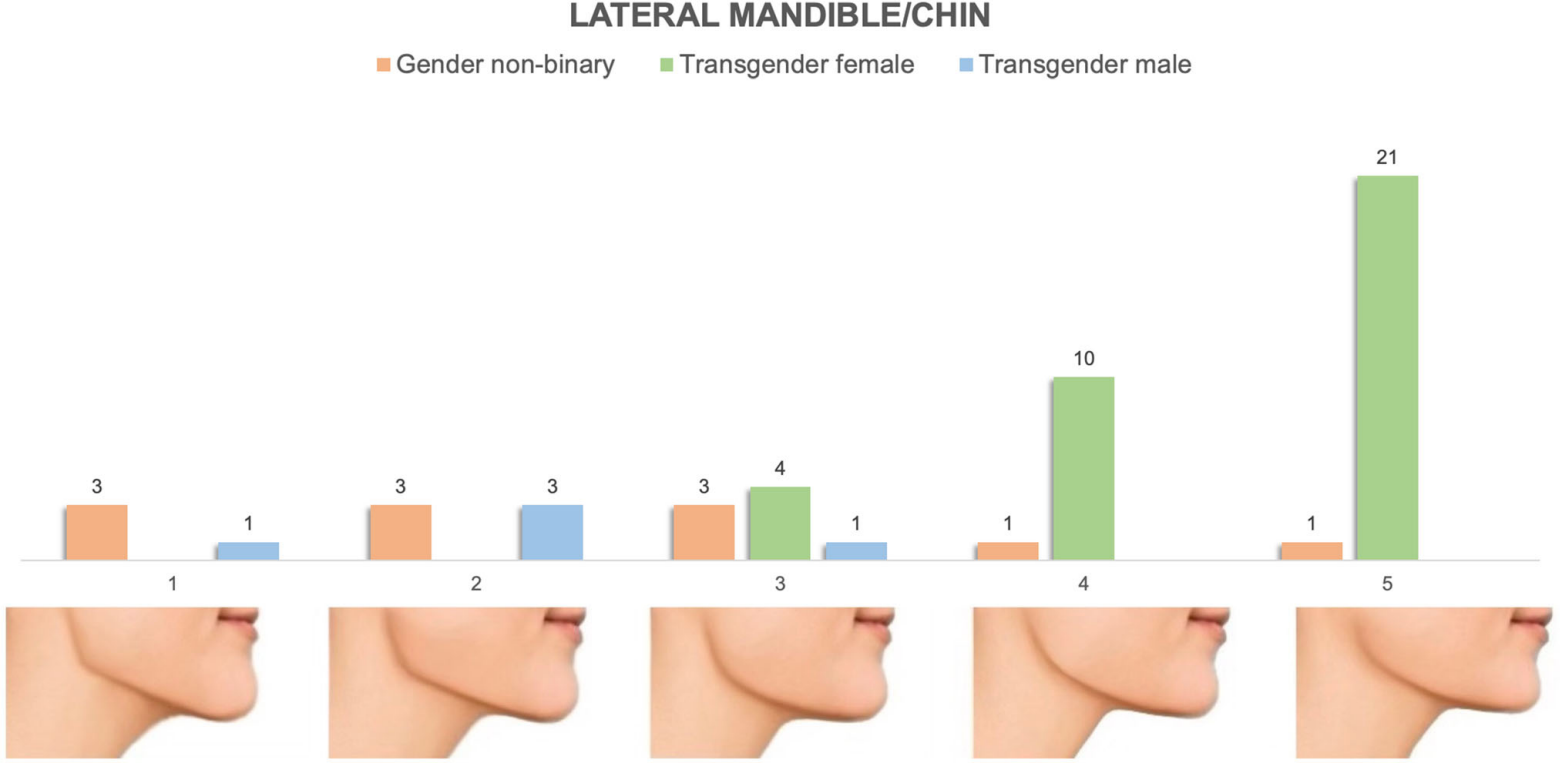
Preferences of lateral mandible/chin appearance stratified by gender identity. It demonstrates tabulated survey responses (stratified and color-coded by gender identity) above their corresponding images, numbered 1–5. On lateral view, our images range from masculine gonial angles (130 degrees), chin height and position to feminine appearing lateral mandible/chin images characterized by decreased chin height, chin position in line with the subnasale and less defined gonial angles of 125 degrees

Bigonial width, chin height/width and lower facial contour are key modifiable areas of gender-conveying lower facial appearance. A survey of craniofacial surgeons aimed to characterize the ideal characteristics of the male gonial angle [18] while multiple prior studies have evaluated aesthetic and masculine/feminine chin position [17, 23, 24]. Across these studies, 130 degrees was reported as the “ideal” male gonial angle with female angles in the 120–125 degree range. Our images span this range of gonial angles with feminine appearing lateral mandible/chin images (Fig. 6, Images 4 + 5) characterized by decreased chin height, chin position in line with the subnasale, and less defined gonial angles of 125 degrees. On frontal view, feminine images were characterized by narrow intergonial and chin width with a soft, heart-shaped contour of the lower face (Fig. 5, Images 4 + 5). Masculine images were characterized by wider and more well-defined gonial angles of 127-130 degrees, taller, wider and more well-defined chins and overall, more angular appearance of the lower face (Figs. 5 and 6, Images 1 + 2). On lateral view, masculine chins were in line with or slightly anterior to subnasale (Fig. 6, Images 1 + 2), while feminine chins were consistently in line with the subnasale (Fig. 6, Images 4+5) [17].

### Data Analysis

Survey response data were tabulated in Qualtrics and analyzed in R-Studio (R-Studio). Categorical data were analyzed using Fisher’s exact tests and continuous variables analyzed using ANOVA with post hoc Bonferroni-adjusted comparisons. Stepwise univariable analysis of age, race, sexual orientation, population setting, income level and gender identity was performed to identify significant predictors of facial preferences (Fisher exact test for categorical variables, univariable linear regression for continuous variables). Significant predictors on univariable analysis (gender identity, all series; age, lateral forehead and lateral mandible series) were then included in a multivariable regression model which revealed only gender identity to be a significant predictor of facial appearance. As a result, data were then stratified by gender identity and compared using Fisher’s exact tests. A *p* value < 0.05 was considered statistically significant.

## Results

### Demographics

A total of 180 patients were identified by chart review and distributed surveys via email. Fifty-nine patients responded, yielding a survey response rate of 32% (58/180).

Patient demographics segregated by self-identified gender identity are shown in Table 1. Transgender male patients comprised the smallest cohort (*n* = 5) followed by nonbinary patients (*n* = 14) and transgender female patients (*n* = 38). These patients tended to be younger than nonbinary or transgender female respondents (25.4 vs. 37.3 vs. 33.6; *p *= 0.06). Nonbinary patients reported lower levels of hormone therapy when compared to transgender male or female patients (35.7 vs. 100%, *p < *0.005). Distributions of race, sexual orientation, population setting and income level were not significantly different among the groups. More than half of our surveyed population reported incomes of less than $50,000 USD per year and only 10% live in a rural setting. All respondents reported undergoing previous gender-affirming surgeries prior to the time of survey completion.

**Table 1:** Respondent demographics stratified by gender identity

	Total cohort (*n* = 58)	TG male (*n* = 5)	TG female (*n* = 39)	Non-binary (*n* = 14)	*p* value
Age (mean, SD)	35.39 (11.3)	25.4 (7.0)	37.3 (12.6)	33.6 (5.7)	0.065
Race/ethnicity (*n*, %)					
White	44 (75.8)	4 (6.8)	29 (52.7)	11 (18.9)	*0.96*
Asian	8 (13.8)	1 (1.7)	6 (10.9)	1 (1.7)	
Black	3 (5.1)	0 (0)	2 (3.4)	1 (1.7)	
Other	3 (5.1)	0 (0)	2 (3.4)	1 (1.7)	
Sex assigned at birth (*n*, %)					
M	41 (70.5)	0 (0)	39 (67.2)	2 (3.4)	< 0.005**
F	17 (29.5)	5 (8.6)	0 (0)	12 (20.6)	
Sexual orientation (*n*, %)					
Asexual	12 (20.6)	0 (0)	0 (0)	1 (1.7)	*0.167*
Bisexual	1 (1.7)	3 (5.2)	14 (24.1)	5 (8.6)	
Heterosexual	22 (37.9)	0 (0)	10 (17.2)	0 (0)	
Lesbian/gay	10 (17.2)	1 (1.7)	9 (15.5)	3 (5.2)	
Other	13 (22.4)	1 (1.7)	6 (10.3)	5 (8.6)	
Region (*n*, %)					
Midwest	4 (6.9)	1 (1.7)	3 (5.2)	0 (0)	*0.094*
Northeast	6 (10.3)	2 (3.4)	2 (3.4)	2 (3.4)	
West	47 (81.0)	2 (3.4)	33 (56.9)	12 (20.6)	
South	1 (1.7)	0 (0)	1 (2.5)	0 (0)	
Population (*n*, %)					
Urban	45 (77.6)	3 (5.2)	29 (50.0)	13 (22.4)	*0.389*
Urban cluster	12 (20.7)	2 (3.4)	9 (15.5)	1 (1.7)	
Rural	1 (1.7)	0 (0)	1 (1.7)	0 (0)	
Income level (*n*, %)					
< 50k	27 (46.5)	2 (3.4)	22 (37.9)	3 (5.2)	*0.63*
50–100k	21 (36.2)	3 (5.2)	11 (18.9)	7 (12.1)	
> 150k	10 (17.2)	0 (0)	6 (10.3)	4 (6.9)	
HRT (*n*, %)					
Yes	53 (91.4)	5 (100)	30 (100)	5 (62.5)	< 0.005**
Hx GAS (*n*, %)					
Any	58 (100)	5 (100)	39 (100)	14 (100)	*NS*
Top	32 (55.2)	5 (100)	15 (38.4)	12 (85.7)	
Bottom	18 (31.0)	0 (0)	17 (43.6)	1 (7.1)	
Face	38 (65.5)	1 (20)	35 (89.7)	2 (14.2)	

### Preferences of Facial Appearance: Survey Responses

Individual distributions of facial preference by cohort and image series are described below.

#### Frontal Forehead and Hairline Appearance

Figures 2 and 3 demonstrate the distribution of preferences of frontal forehead and oblique hairline appearances stratified by gender identity. Transgender female patients preferentially selected "feminine" images 4 and 5 on both frontal forehead [Image 4 (*n* = 14, 40%), Image 5 (*n* = 11, 31%)]. and oblique hairline series [Image 4 (*n* = 19, 56%), Image 5 (*n* = 6, 18%)]. 80% (*n* = 4) of transgender male patients selected the most masculine appearing forehead on oblique view, while their preferences were more varied on frontal view, ranging from M-shaped [Image 1 (*n* = 2, 40%)] to short triangular [Image 5 (*n* = 1, 20%)]. Nonbinary patients tended to identify more masculine appearing forehead and hairline preferences, especially on the frontal view [Image 2 (*n *= 7, 63%)], but had varied preferences on oblique view.

#### Lateral Forehead/Nasofrontal Complex Appearance

Figure 4 demonstrates the distribution of preferences of lateral forehead/nasofrontal complex appearance stratified by gender identity. Transgender females strongly selected more feminine images characterized by more obtuse nasofrontal angles (135 degrees: *n *= 16, 46%; 139 degrees: *n* = 13, 37%), decreased forehead inclination (− 4 degrees: *n* = 16, 46%; − 3 degrees: *n* = 13, 37%) and no frontal bossing (*n* = 29, 83%). Both nonbinary and transgender male patients had no clear distribution of masculine versus feminine selections. Only three patients in any cohort preferred the most masculine nasofrontal complex depiction.

#### Mandible/Chin Appearance

Figures 5 and 6 demonstrate the distribution of preferences of frontal and lateral mandible/chin appearance stratified by gender identity. Transgender females preferred feminine appearing lateral mandible/chin images characterized by decreased chin height, mandibular prominence and less defined gonial angles [Image 4 (*n* = 10, 29%), Image 5 (*n* = 21, 60%)]. On frontal view, transgender female patients identified a similar distribution of feminine appearing images, preferentially selecting those characterized by narrow intergonial and chin width with a soft, heart-shaped contour of the lower face [Image 4 (*n* = 14, 40%), Image 5 (*n* = 16, 46%)]. Nonbinary and transgender male patients had varied selections in both frontal and lateral mandible/chin series.

### Preferences of Facial Appearance: Subset Analysis and Multivariable Regression

Univariable analysis demonstrated gender identity to be associated with facial preferences across all series, while age was found to be associated with facial preferences in the lateral forehead and lateral mandible image series. Inclusion of age and gender identity in a multivariable linear regression model demonstrated only gender identity to be significantly associated with preferences of facial appearance (Table 2). As a result, data were then segregated by gender identity and compared using Fisher’s exact tests. Transgender female patients had significantly different preferences when compared to transgender male and gender nonbinary patients across all image series (*p* < 0.005 for all comparisons and series). There was no statistically significantly difference when comparing the distribution of preferences reported by transgender male and nonbinary patients (*p* > 0.05 for all comparisons and series).

**Table 2:** Multivariable linear regression for facial preferences

Predictors	Lateral forehead		Lateral mandible	
	*R*^2^ = 0.4303 *F* (3, 47) = 13.59 *p* < 0.0001		*R*^2^ = 0.5769 *F* (3, 47) = 23.72 *p* < 0.0001	
	*β*	*p*	*β*	*p*
Intercept	2.168	< 0.001	2.167	< 0.001
Age	0.016	0.159	0.008	0.439
Transgender male	− 0.594	0.243	− 0.386	0.419
Transgender female	1.377	< 0.001	1.996	< 0.001

### Preferences of Facial Appearance: Subset Analysis of Nonbinary Patients

**Fig. 7:**
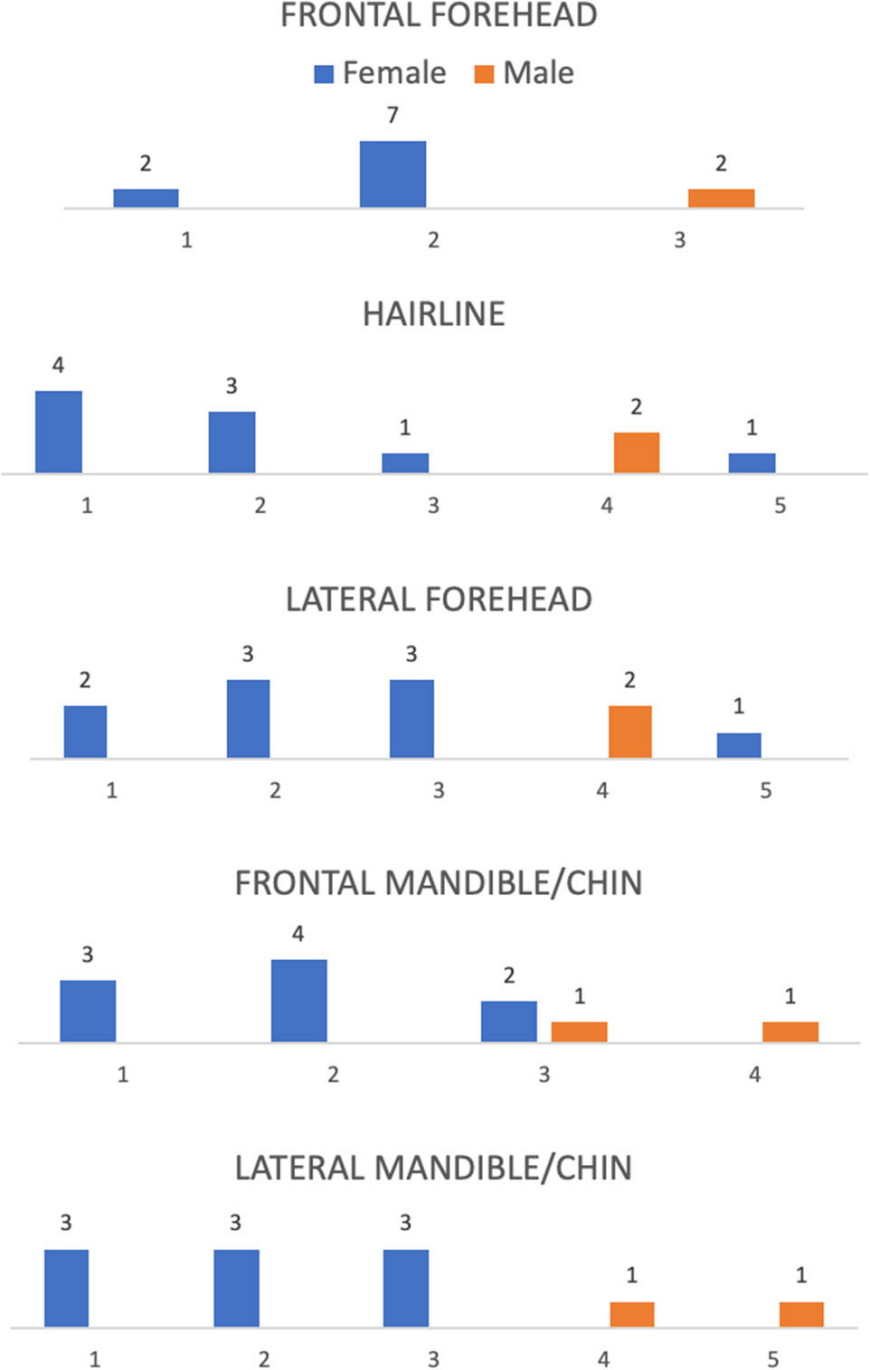
Nonbinary patient preferences of facial appearance on all image series stratified by assigned sex at birth. Survey responses of only gender nonbinary participants are shown above. Responses are color coded/stratified by assigned sex at birth and displayed as separate bar graphs for each image series. The corresponding image numbers are displayed on the *X*-axis, however the series images themselves are not shown for purposes of brevity

Figure 7 demonstrates the distribution of preferences of nonbinary patients for all series stratified by sex assigned at birth. Assigned sex at birth was significantly associated with preferences across all series (*p* < 0.05) aside from the frontal mandible series (*p* = 0.21). Respondents consistently identified preferences neutral to or opposite of their assigned sex at birth in 53 of 55 selections.

### Perspectives on Preoperative Planning and Simulation

78.4% (40/51) of respondents indicated that having similar types of image series available for preoperative planning discussions or a digital app that could simulate planned operative changes prior to gender affirming facial surgery would be "very" (12/51) or "extremely" useful (28/51). There was a similar degree of enthusiasm for such resources between cohorts (*p* > 0.05).

## Discussion

FGAS is a psychosocially critical intervention for patients with gender dysphoria [25, 26] yet little data exists from the perspective of transgender, and in particular, nonbinary patients, regarding their goals for facial appearance following these procedures.

Given that FGAS focuses on alleviating dysphoria with various facial features by transforming facial anatomy, it is essential to tailor these results accordingly [27]. Ascertaining the preferences of gender nonbinary patients will help better elucidate key elements of FGAS in this population and better prepare surgeons for preoperative consultation and surgical planning. As a result, we performed a cross-sectional survey of transgender and gender nonbinary patients utilizing visual analog scales derived from published anthropometric data. Our survey represents the first examination of preferences of facial appearance in transgender and nonbinary patients in regard to FGAS.

Not unexpectedly, we found that transgender female patients significantly preferred more feminine features than transgender male and nonbinary patients. In contrast, nonbinary and transgender male patients identified varied preferences without clear distribution towards masculine or feminine features across all facial regions. When stratified by assigned sex at birth, nonbinary patient preferences aligned with facial features opposite those of their assigned sex. Overall, TM participants had the least predictable preferences of all cohorts.

### Preferences of Facial Appearance

#### Nasofrontal Region

The nasofrontal complex represents a key area in regard to characterizing masculine or feminine appearance with well-described nasofrontal angle, frontal bossing and forehead inclination patterns in both male and female skulls [1–3, 6, 9, 15]. In our study, transgender females indicated preferences for obtuse nasofrontal angles of 132–139 degrees in conjunction with minimal frontal bossing and forehead retroclination of − 3 to − 7 degrees consistent with previously published descriptions of classically feminine anthropometrics [2, 3, 13, 16]. TM and nonbinary patients, however, had varied preferences encompassing the full spectrum of features. When stratified by sex assigned at birth, nonbinary patients preferences segregated more clearly, with all assigned males preferring feminine Image 4 (nasofrontal angle of 135 degrees, no frontal bossing, − 4 degree forehead retroclination), and nearly 91% of patients assigned female at birth preferring masculine images 1–3 (nasofrontal angles of 125–132 degrees, with moderate to severe bossing and mild to significant forehead retroclination). Interestingly, TM patients identified a similarly broad range of preferences.

Prior investigations of preferences of facial appearance specifically in the transgender and gender nonbinary patient population are limited. Ching et al. surveyed both transgender and cisgender patients as well as surgeons performing FFS in regard to the attractiveness and femininity of various iterations of supratip break, nasal tip width and gonial angle [29]. Within these facial regions, they reported that TF trended towards selecting more feminine-appearing images than cisgender females. Interestingly, surgeons also selected images in this fashion. They posited that this trend perhaps indicated a preference for "hyperfeminine" appearance (by both TF respondents and surgeons) to ensure social perception of patients’ identified gender [29]. A high proportion of TF respondents in our study selected the nasofrontal complex image with the most feminine features, which may suggest a similar tendency. TM patients lacked equivalent preference for "hypermasculine" facial features, which may be artifact due to a small sample size or may indicate an overall preference for non-feminine features without strong preference for explicitly masculine ones. Interestingly, only four patients selected the most masculine appearing image (Fig. 4 Image 1) which may also suggest a general perception of unattractiveness of the combination of marked bossing, aggressive forehead retroclination and acute nasofrontal angle in this image.

#### Frontal Forehead and Hairline

Aside from the nasofrontal complex, forehead shape and hairline combine to form a second element contributing to the gendered appearance of the upper third of the face [30]. The preferences elected by TM and TF participants in our study followed those that would be expected with self-identified masculine/feminine features of gender identity. TM respondents elected predominantly masculine or neutral appearing forehead shape and hairline patterns (“M” shaped with tall broad non-hair bearing forehead and frontotemporal recession) while TF respondents chose shorter and less broad non-hair bearing forehead length and more feminine hairline patterns (“round” and “bell” shaped on frontal view, with the presence of temporal points and no fronto-temporal recession). Nonbinary patients again represented a spectrum of preferences encompassing the entire range of appearances in the forehead and hairline shape series that aligned when segregated by sex assigned at birth in 10 of 11 patients (Fig. 7). While our data demonstrated consistent segregation among TM, TF and assigned sex-stratified nonbinary patients with regard to hairline pattern preference, hairline patterns are variable even within cohorts of equivalent gender and race [19–21]. As a result, preferences for hairline pattern may be influenced by respondent baseline characteristics and their impact on gender identity related dysphoria may be more variable than other facial features.

In contrast to surgical management of the nasofrontal complex, maneuvers for manipulation of the hairline position and forehead shape are less nuanced. Pretrichial incisions are commonly used in facial feminization to lower the hairline and reduced non-hair bearing vertical forehead height [31–33]. However, the classically male phenotype of temporal recession and wide forehead are challenging to address with browlift incisions alone [31–33]. More recent studies demonstrate the powerful effect of targeted augmentation of the temporal recession in facial feminization patients using protocols for simultaneous hair transplantation and frontal bone setback [30]. Advances in hair transplantation techniques have allowed more specific control of hairline patterns and will likely play a growing role in the management of the forehead and hairline in FGAS [34]. As a result, forehead shape and hairline contour will be important elements to discuss with all patients presenting for consultation regarding FGAS.

#### Mandible and Chin

Preferences of our cohort regarding the lower face mirrored the trends observed in the nasofrontal complex. Transgender females significantly preferred classically feminine features of the mandible (soft gonial angles, narrow intergonial width) and chin (narrow, short chins) that yield an overall "heart-shaped" contour of the lower face. Nonbinary and TM patient preferences again encompassed the full range of masculine to feminine features. In the case of nonbinary patients, these preferences stratified clearly by assigned sex at birth.

Our results regarding preferences of TFs align with findings by Ching et al that narrower gonial angles were identified as more attractive [29]. Similar to the patterns of preferences solicited regarding the nasofrontal complex, TF but not TM appeared to preferentially elect "hyperfeminine" images, with TM opting for neutral/less masculine options.

Surgical control of the lower face includes feminization of the masculine mandible via gonial angle reduction through burring and ostectomy as well as reduction genioplasty via burring or wedge osteotomies [10]. Overlying soft tissues and masseteric muscle bulk play a role in appearance as well, however, aside from limitations imposed by location of neurovascular structures, craniofacial manipulation allows fairly custom control of the mandibular appearance that can be well tailored to individual patient preferences. These procedures, as well as non-surgical interventions [28], are already a well described aspect of facial feminization surgery, but here we highlight the importance of tailoring these maneuvers based on individualized preoperative planning with nonbinary and transgender male patients.

### Preferences of Gender Nonbinary and Transgender Male Patients

As hypothesized, nonbinary patients in our study reported varied preferences that failed to segregate clearly like those of transgender females. Interestingly, stratifying these patients by sex assigned at birth revealed that patients consistently preferred features associated with the gender identity opposite to their assigned sex. This, in part, explains the visually observable trend that nonbinary patients selected more masculine features in all series across Fig. 1 than transgender male patients. Further, nonbinary, assigned-female-at-birth respondents selected more masculine images than those of their transgender male counterparts. Subsequently, our data suggests that transgender male patients reported preferences that were least predictable among the cohorts. Interestingly, this may explain why the incidence of TM patients seeking FAGS is lower than nonbinary and TF patients within our cohort.

This trend may represent a preference for a more neutral facial appearance without strong gender identifying features in either direction [35]. The motivation behind this preference, based on a qualitative analyses of gender dysphoria in nonbinary patients, may be driven mainly by fears of unwanted gender assumptions by others associated with such features [35]. It may also indicate a disinterest or lack of priority of the importance of facial gender conveying features or “shifting dysphoria [35]. Empirically derived explanations for this trend are limited given the paucity of literature regarding goals of nonbinary and transgender male patients in gender affirming surgery. Only the aforementioned study by Ching et al has touched on nonbinary preferences regarding facial appearance but was limited to a social media-based nonbinary cohort of four patients and perspectives of only the nasal width, tip projection/profile and gonial angle [29]. Further, preferences were not stratified by gender identity, and as a result, no information is available regarding specifically the nonbinary cohort of that study. Future prospective studies with larger cohorts and patient-reported outcome measures are needed to confirm these initial findings.

### Preoperative Simulation

Respondents across all three cohorts indicated a high desirability of visual analog scale tools for preoperative simulation or counseling. As AI and facial recognition software improves, the accuracy and accessibility of facial manipulation tools continues to grow [36–40]. We foresee these technologies playing a key role in preoperative planning and counseling for patients moving forward in FGAS [41]. Opportunities for future studies include examining these tools to streamline preoperative surgical consultations as well establish realistic patient expectations for postoperative outcomes.

### Limitations and Future Directions

Our study is subject to a number of limitations including those associated with objective assessment of subjective measures, retrospective and survey-based study design, and cohort size.

Querying participants about preferences of appearance is inherently challenging. Issues with visual analog scales or rating systems in this area have been well described in other studies [42]. We attempted to provide realistic yet well controlled image series and minimize pitfalls of visual rating analogs/scales. To guide the accuracy of our data series, we derived objective measurements within our images from published anthropometric data. To represent a spectrum of masculine and feminine features, we set either extreme of the image series as the 95% CI of various measures and then spanned the range between the two using standard deviation of published data when available. Further, we linked responses to a visual analog scale (shown to yield higher reliability by Alford et al) and displayed image series side by side as a reference that would be consistent across all respondents [42]. Further, these images held all features aside from those in question constant, eliminating the influence of other photographic elements that may confound answers. While limiting exposure to one facial feature isolates impact of that feature, the harmony between various facial regions has been shown to impact preferences or perceptions of individual features [27, 43]. As a result, it may have been beneficial to include a series of composite features where all change simultaneously, though this would have significantly increased survey length and decreased likelihood of survey completion. To ensure consistency in image generation and editing, we used images that were mostly of the same ethnicity. However, it is worth noting that the ethnicity of the images used may have influenced the survey responses of participants with different ethnic backgrounds.

Although we achieved a reasonable survey response rate (32%), the majority of respondents were TF with a moderate population of nonbinary patients and a limited group of TM respondents. By virtue of its cross-sectional, survey-based nature, our study is also subject to limitations shared by all survey-based studies such as response bias. Additionally, many TF respondents had already undergone FFS at our institution, and as a result, their preoperative counseling may have impacted how they evaluate facial appearance in the setting of FGAS. Finally, we elected to focus our investigation on bony craniofacial skeletal elements addressed in FGAS, however, nasal appearance and soft tissues of the midface also contribute to facial gender appearance but were not explored in our survey. We elected to omit these areas as they are extensively described elsewhere in the literature.

## Conclusions

Nonbinary patients have a spectrum of preferences regarding facial appearance that do not align with those of binary transgender males/females. While nonbinary patient preferences tend to be those opposite of their assigned sex at birth, transgender male patients have variable preferences. With clear preoperative goals and expectations, FGAS can yield excellent postoperative results—both aesthetic and psychosocial. As such, our findings represent important information for the surgeon in preoperative counseling and assessment with these patients to achieve the most successful and targeted outcomes for management of dysphoria secondary to facial characteristics.
